# Appendicitis Post Fall in the Pediatric Population: A Case Report

**DOI:** 10.7759/cureus.49603

**Published:** 2023-11-28

**Authors:** Nouf Albalawi, Mishal Alhazmi, Abduallah ALqahtani, Abdullah Aloboudi, Alanoud Mesawa, Naif Alotaibi, Amel Babiker

**Affiliations:** 1 Pediatric Emergency Medicine, King Abdullah Specialized Children's Hospital, Riyadh, SAU; 2 Pediatric Radiology, King Abdullah Specialized Children's Hospital, Riyadh, SAU; 3 Emergency Medicine, King Abdulaziz Medical City, Riyadh, SAU

**Keywords:** blunt trauma, right abdominal pain, fall down, appendicitis, trauma

## Abstract

The appendix is a small, finger-sized tubular pouch that extends from your large intestine. Regardless, the physiology of the appendix is still unclear. There are several factors that cause appendicitis, such as infection, underlying tumor, constipation, and trauma. Symptoms of right lower quadrant abdominal pain, nausea, anorexia, and fever, as well as physical signs such as rebound tenderness and McBurney point/sign, are typical of non-traumatic acute appendicitis. On the other hand, a thorough history and physical examination are paramount for an accurate diagnosis of acute traumatic appendicitis. If the history and physical examination are inconclusive, further evaluation with ultrasonography or computed tomography (CT) is advised. Upon reaching an accurate diagnosis, the course of treatment involves an appendectomy and intravenous antibiotics. This case describes a patient who suffered blunt traumatic abdominal injury from a fall resulting in acute appendicitis. For pediatric patients who complain of abdominal pain and present to the emergency department (ED), appendicitis should be on the differential diagnosis list, even if the patient's symptoms started after blunt abdominal trauma. Due to the rarity of appendicitis after trauma, rapid identification necessitates a high index of suspicion.

## Introduction

Appendicitis is the inflammation of the appendix, which attaches to a hollow organ in the upper part of the cecum, usually in the lower right part of the abdomen [1]. Subsequently, the most common cause for surgical consultation in the emergency department (ED) is acute appendicitis [2]. In both adults and children, it is one of the most typical causes of sudden, severe abdominal pain. The diagnosis of acute appendicitis in a young patient is still a challenge, as most of these patients show up late [3].

After all, the complications of appendicitis include perforation leading to an abscess, systemic peritonitis, and sepsis [4]. Over 50,000 and 300,000 acute appendicectomies are performed annually in the United Kingdom and the United States [4]. Respectively, it makes acute appendicitis the most frequent abdominal surgical emergency globally. The incidence of appendicitis is still unknown [5].

## Case presentation

**Figure 1:**
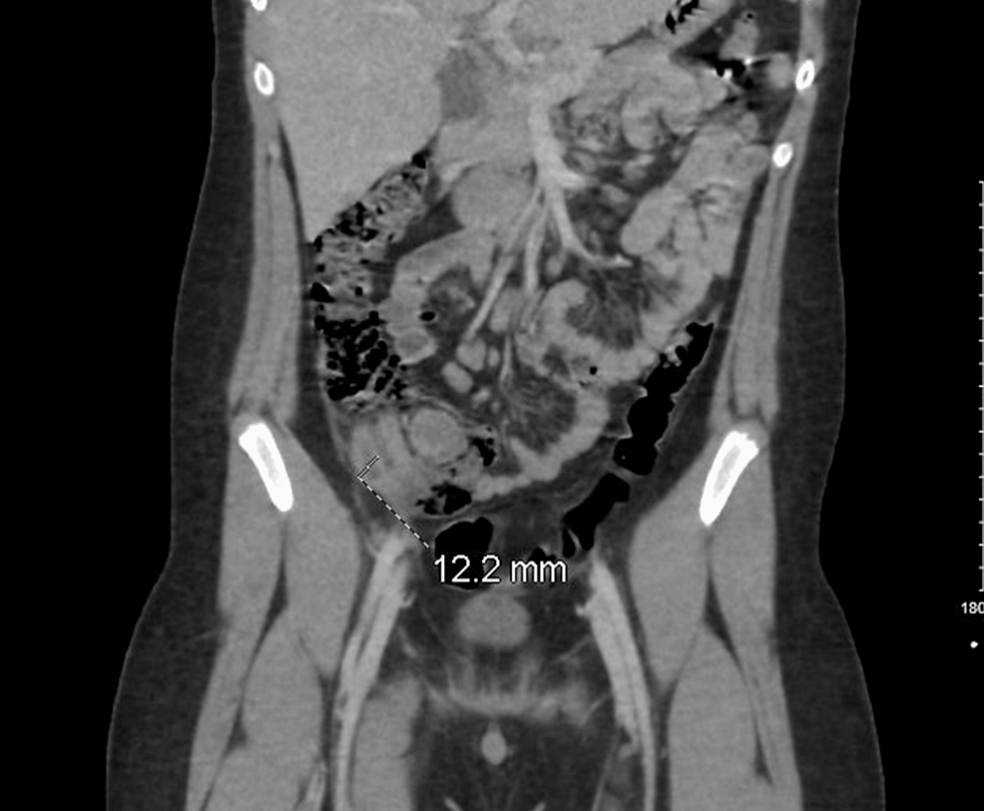
The appendix is seen in the right lower quadrant measuring up to 12.2 mm (1.2 cm) in the maximum diameter with surrounding fat stranding and several prominent mesenteric lymph nodes.

A 10-year-old male, with no medical history of illness or operation, presented to the emergency department (ED) with complaints of lower abdominal pain that started one day ago, after he tripped and fell from a 2 m height when he was jumping on a trampoline. Soon after he fell, on the right side of the abdomen as per the patient, there was mild lower abdominal pain that increased in severity, which necessitated his visit to the ED. Upon presentation, the patient was fully conscious, his Glasgow Coma Scale (GCS) was 15/15, he was vitally stable, and he denies any loss of consciousness, headache, or vomiting. The physical examination was negative for palpable skull fractures, rhinorrhea, otorrhea, and bruising in the face or abdominal wall. Also, there is no hemotympanum or septal hematoma; a focused examination of the abdomen showed that it was soft and lax with moderate tenderness over the suprapubic area and right lower quadrant with positive rebound tenderness. In addition, positive bowel sounds were documented, and bedside focused assessment with sonography in trauma (FAST) was negative. Meanwhile, his laboratory results showed a white blood cell (WBC) count of 12.30 × 10^9^/L with an absolute neutrophil count of 7.47-12.30 × 10^9^/L, around 60.9% of total WBC. His liver enzyme, renal profile, pancreatic enzyme, coagulation profile, lactic acid, and electrolytes were within normal range (Table 1). The abdomen and pelvis computed tomography (CT) showed features of uncomplicated acute appendicitis with multiple mesenteric lymphadenopathy (Figure 1).

**Table 1:** Laboratory results WBC, white blood cell; Hgb, hemoglobin; INR, international normalized ratio; PT, prothrombin time; PTT, partial thromboplastin time; ALT, alanine transaminase; AST, aspartate transaminase; BUN, blood urea nitrogen

Laboratory parameters	Result	Reference range
WBC	12.30	4-12 × 10^9^/L
Hgb	134	113-150 g/L
RBC	4.90	4.35-5.5 × 10^12^/L
Platelets	323	150-450 × 10^9^/L
Neutrophils	7.47	2-7.4 × 10^9^/L
Lymphocytes	2.80	1.5-4.5 × 10^9^/L
Monocytes	1.33	0.1-2.3 × 10^9^/L
Eosinophils	0.57	0.1-1.6 × 10^9^/L
INR	0.98	0.8-1.2
PT	10.70	9.38-12.34 seconds
PTT	28.20	24.84-32.96 seconds
ALT	16	5-55 U/L
AST	17	5-34 U/L
Total bilirubin	4.8	~20.5 µmol/L
Creatinine	55	27-62 µmol/L
BUN	4.8	2.5-6 mmol/L
CO_2_	24	23-30 mEq/L
Potassium	4.7	3.4-4.7 mmol/L
Sodium	138	135-145 mEq/L
Chloride	108	98-106 mEq/L
Glucose random	6.1	2.2-3.3 mmol/L

The pediatric surgeon was involved in the management; they admitted the patient for analgesia, antibiotics, and observation. They kept the patient for observation for two days, throughout which he was clinically and vitally stable with dissipating abdominal pain. He tolerated his meals very well without complication and passed normal bowel motions. Examination at the time showed no more tenderness or rebound tenderness. Finally, the patient was discharged from the hospital with full recovery without the need for further follow-up.

## Discussion

The pediatric population is at a greater risk than adults for intra-abdominal injuries following blunt abdominal trauma, and this is related to multiple factors, including having an immature musculoskeletal system and weaker abdominal wall musculature than adults. However, the abdominal cavity of children is smaller and less protective of underlying structures, so a given force directed to the abdomen will be distributed over the smaller body surface area of the child, increasing the likelihood of injury to the underlying organs [5]. Most cases of appendicitis, traumatic or non-traumatic, are predominantly male, and blunt trauma is common in males in this age group, as our patient [6].

The appendicitis pathophysiology is the obstruction of its lumen by stool, foreign bodies, parasites, etc. or the indirect effects that cause mucosal injury, edema, or hematoma following blunt trauma, resulting in the narrowing of the lumen, obstruction, and inflammation [7]. Although there are limited reports about the relationship between blunt abdominal trauma and appendicitis, some theories support it [7]. Acute appendicitis has been reported in a spectrum of pediatric age groups as a result of blunt abdominal injuries. Among these reported cases, the presentation of traumatic appendicitis resembles that of non-traumatic appendicitis, with right lower quadrant abdominal tenderness, nausea, and anorexia [8]. Several factors suggested appendicitis secondary to blunt abdominal trauma [9], such as the presence of abdominal pain post trauma that was not present before trauma [5,10]. In the literature, patients present with appendicitis after severe blunt trauma directed to the abdomen for 6-48 hours, as our patient did [10,11].

Unfortunately, the number of blunt trauma-induced appendicitis cases reported in the literature is lacking. In one study, the number of cases documented is around 45 cases and merely 16 of which are reported in children [12,5,11]. The lack of publications in this regard, especially in the pediatric population, limits the proper assessment of the scope of the disease. Despite acute appendicitis being the leading cause of surgery of the abdomen in the ED, the incidence of traumatic appendicitis remains low. In another study, 48 cases of trauma-induced appendicitis were reported out of 13,496 cases of appendicitis (0.3%) and only five cases out of 554 patients (0.9%) of post blunt trauma [8].

To reach an accurate definition of trauma-induced appendicitis, several strict conditions should be entertained to reach traumatic appendicitis [13], an unremarkable previous attack of appendicitis. The causative objective and mechanism of trauma must be a direct, blunt, violent force reaching the appendix. The effects of the trauma must be experienced immediately and merged with those of acute appendicitis shortly after the trauma. The appendix must be operatively demonstrated as a true traumatic lesion. There must be a superimposed acute inflammation of the appendix, the result of the traumatic lesion, to confirm the diagnosis microscopically and no evidence of chronic pathology.

Finally, in trauma-induced appendicitis, a sudden increase in intra-abdominal pressure is believed to be transmitted to the cecum or obstruction, hemorrhage, and perforation of the appendiceal lumen [14]. Furthermore, the exact mechanism is still unclear, despite the reported cases of post-traumatic appendicitis that had no radiological evidence of an appendicolith. A careful assessment is mandated, and a high index of suspicion is required to diagnose trauma-induced appendicitis.

## Conclusions

Appendicitis post blunt trauma is very rare, although any patient who presents to the ER with a history of abdominal pain, especially in the lower or right lower quadrant, should raise suspicion for appendicitis. However, the coexistence of appendicitis with trauma raises a clinical dilemma of whether the trauma is the culprit causing appendicitis or not.
